# Is podocytopathy another image of renal affection in p-SLE?

**DOI:** 10.1186/s12969-021-00547-9

**Published:** 2021-04-26

**Authors:** Hend H. Abdelnabi

**Affiliations:** Nephrology Unit, Pediatric Department, Tanta University, El-Geesh Street, Tanta, Egypt

**Keywords:** Pediatric systemic lupus erythematosus, Podocytopathy, Lupus nephritis

## Abstract

**Background:**

Lupus podocytopathy (LP) is a renal affection described in systemic lupus erythematosus (SLE) patients with nephrotic range proteinuria, characterized by diffuse foot process effacement without immune deposits and glomerular proliferation. This study describes LP, its pathological features and outcomes of pediatric (p-SLE) patients in comparison to the usual lupus nephritis (LN) cases.

**Methodology:**

A retrospective cohort study conducted on a 10-year registration (2010–2019) of 140 p-SLE patients at the Pediatric Department, Tanta University. Histopathological analysis with light microscopy (LM) and immunofluorescence (IF) of all renal biopsies were evaluated according to the International Society of Nephrology Renal Pathology Society (ISN/RPS) grading system. In addition, some biopsies were examined with electron microscopy (EM).

**Results:**

Eighty-six p-SLE cases (61.4%) had renal involvement; seventy-nine biopsies (91.86%) of them met the classification criteria of LN as defined by ISN/RPS system. Five biopsies were normal (MCD) and two showed focal segmental sclerosis (FSGN) that did not meet any known classification of LN. Hence, they were reevaluated using EM that revealed diffuse effaced podocytes without glomerular sub-epithelial, endocapillary or basement membrane immune deposits, and were classified as having lupus podocytopathy, representing (8.14%) of all LN biopsies. Those seven cases showed good response to steroids with a complete remission duration of 3.40 ± 1.95 weeks. However, some case had 1–3 relapses during the duration of follow up.

**Conclusions:**

LP is a spectrum of p-SLE, not an association as it is related to disease activity and its initial presentation.

## Introduction

Systemic lupus erythematosus (SLE) is an inflammatory autoimmune disease affecting multi-systems with periods of activities and remissions [1, 2]. About 15 to 20% of all SLE patients have a disease onset before the age of 16 years (p-SLE), and 60% of cases have lupus nephritis (LN) at early disease onset [3, 4]. The classification of LN revised by the International Society of Nephrology and the Renal Pathology Society (ISN/RPS) in 2018, emphasized that immune complex aggregation is central to LN and universally present in all subclasses [5]. The increasingly recognized phenomenon of apparent minimal change disease (MCD) and focal segmental glomerular sclerosis (FSGS) without significant immune deposits in a patient with SLE is termed lupus podocytopathy (LP) [6]. The diagnosis of LP in a patient with SLE and nephrotic syndrome is based on the finding of diffuse foot process effacement in the absence of peripheral capillary wall immune deposits [7]. LP is not included in the current ISN/RPS classification. Therefore, we aimed in this study to evaluate different histopathological patterns of LN and prevalence of LP, in an attempt to clarify its association with disease activity.

## Methodology

This is a retrospective cohort study conducted on a 10-year registration (2010–2019) of 140 p-SLE patients at the Pediatric Department, Tanta University. The revised 1997 American College of Rheumatology (ACR) criteria was used to establish SLE diagnosis in studied children [8]. The patient’s age at disease onset, sex, clinical symptoms and manifestations at the time of initial presentation and routine investigations [CBC, ESR, serum creatinine and blood urea, 24-h urinary proteins, serum C3 and C4, antinuclear anti-bodies (ANA), anti-double-stranded DNA (anti-dsDNA)] were recorded. SLE Disease Activity Index (SLEDAI) and renal SLEDAI were used to assess the global disease and renal activity respectively [9]. Histopathological analysis of 86 renal biopsies was done in children presented with renal affection at initial diagnosis and evaluated according to the International Society of Nephrology Renal Pathology Society (ISN/RPS) grading system [10]. All biopsies were examined using light microscopy (LM) and immunofluorescence microscopy (IF). In this study, all the cases found to have normal LM examination and did not meet the ISN/RPS classification were examined with EM. Group I (LN) “79 biopsies” had one of the histopathological classifications of LN. Group II (LP) “7 biopsies” did not meet ISN/RPS classification of LN, five of them were normal and two showed focal segmental sclerosis (FSGN), hence they were reevaluated using EM examination to document the presence of any glomerular sub-epithelial, endocapillary or basement membrane immune deposits. *Our treatment regimen;* LN class I and II were treated using hydroxychloroquine (5 mg/kg/d), steroids (0.5-2 mg/kg/d) and other immunosuppressants according to the extrarenal manifestations. LN class III, IV, III/IV + V, V and LP (nephrotic syndrome) received high-dose steroids (2 mg/kg/d), Mycophenolate Mofetil (MMF) (1200 mg/m^2^/d) combined with angiotensin converting enzyme (ACE) inhibitors and hydroxychloroquine for 4 weeks then steroids were tapered to (5 mg/day) after 6 months (Induction phase). This regimen was continued for another 18 months (Maintenance phase). A pulse dose of methylprednisolone (500 mg/m^2^) was given for 5 days in severe cases (neurolupus, vasculitis, renal failure). If complete remission (proteinuria reduction < 500 mg/day, normalized GFR and serum creatinine with controlled blood pressure) was not achieved after 6 months of follow up, we shift MMF to cyclophosphamide (CYC) (500 mg/m^2^/4w) for 6 doses, then once every 3 months for another 18 months. In case of failure to induce remission and in LP cases with FSGS, we give cyclosporine (CsA) (5 mg/kg/d) as a second line in addition to steroids or augmentation regimen (Steroids +MMF + CsA). Lastly, Rituximab is resorted to in resistant cases.

### Statistics

Analysis of data was performed using SPSS statistical software version 21. Qualitative data described using number and percent (n, %). The Kolmogorov-Smirnov test was used to verify the normality of distribution. Quantitative data was described using mean and standard deviation (Mean ± SD), median and interquartile range (Median ± IQR). Chi-Square test (X^2^), Student t-test (t) and Mann Whitney test (U) were used to compare data between studied groups. Cox regression was used to compare the time of event occurrence (complete remission).

## Results

**Fig. 1:**
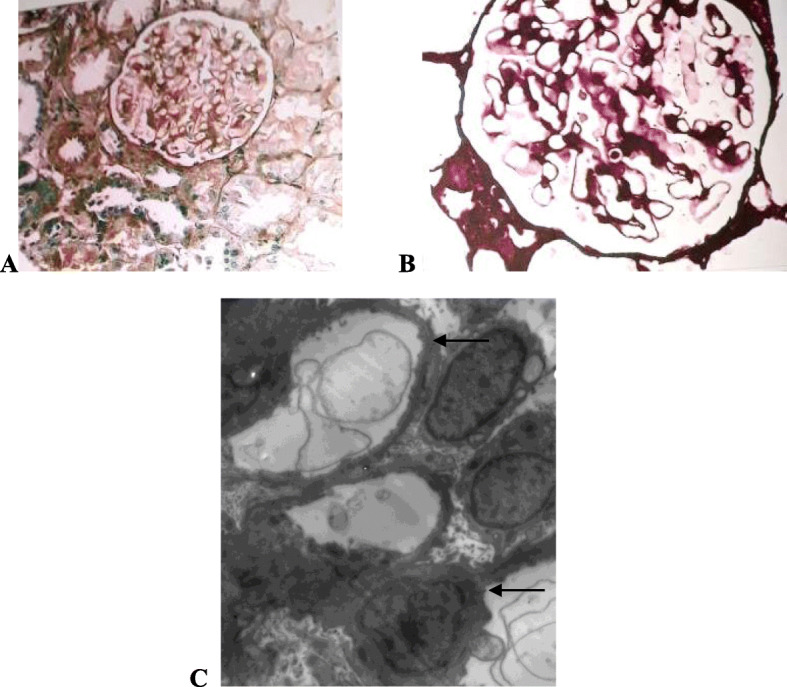
(LM and EM renal biopsy of p-SLE female patient aged 15y presented with massive edema and nephrotic range proteinuria after 2 months of disease diagnosis). **a** LM (H&E), there is no mesangial thickening, normal basement membrane, no hypercellualrity. **b** LM (silver stain) does not show any spikes or vacuolization (no active or chronic. glomerular lesions of lupus nephritis).**c** EM (Mag × 2500) shows diffuse effaced podocytes (black arrows) without any immunological deposits

**Fig. 2:**
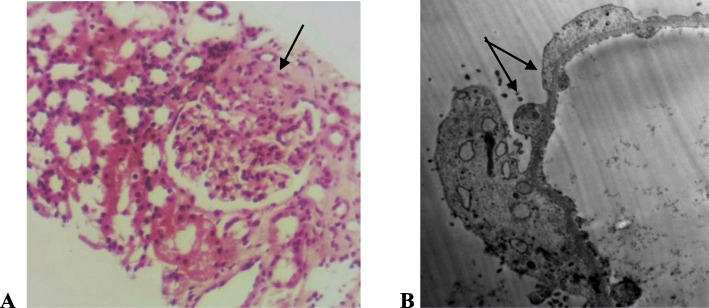
(LM and EM renal biopsy of p-SLE female patient aged 14y presented with weight loss, fever, pericardial effusion, edema, proteinuria and renal impairment at initial disease presentation). **a** LM (H&E) shows FSGS (black arrow). **b** EM (Mag × 2500) shows diffuse effaced podocytes (black arrows) without any immunological deposits

**Fig. 3:**
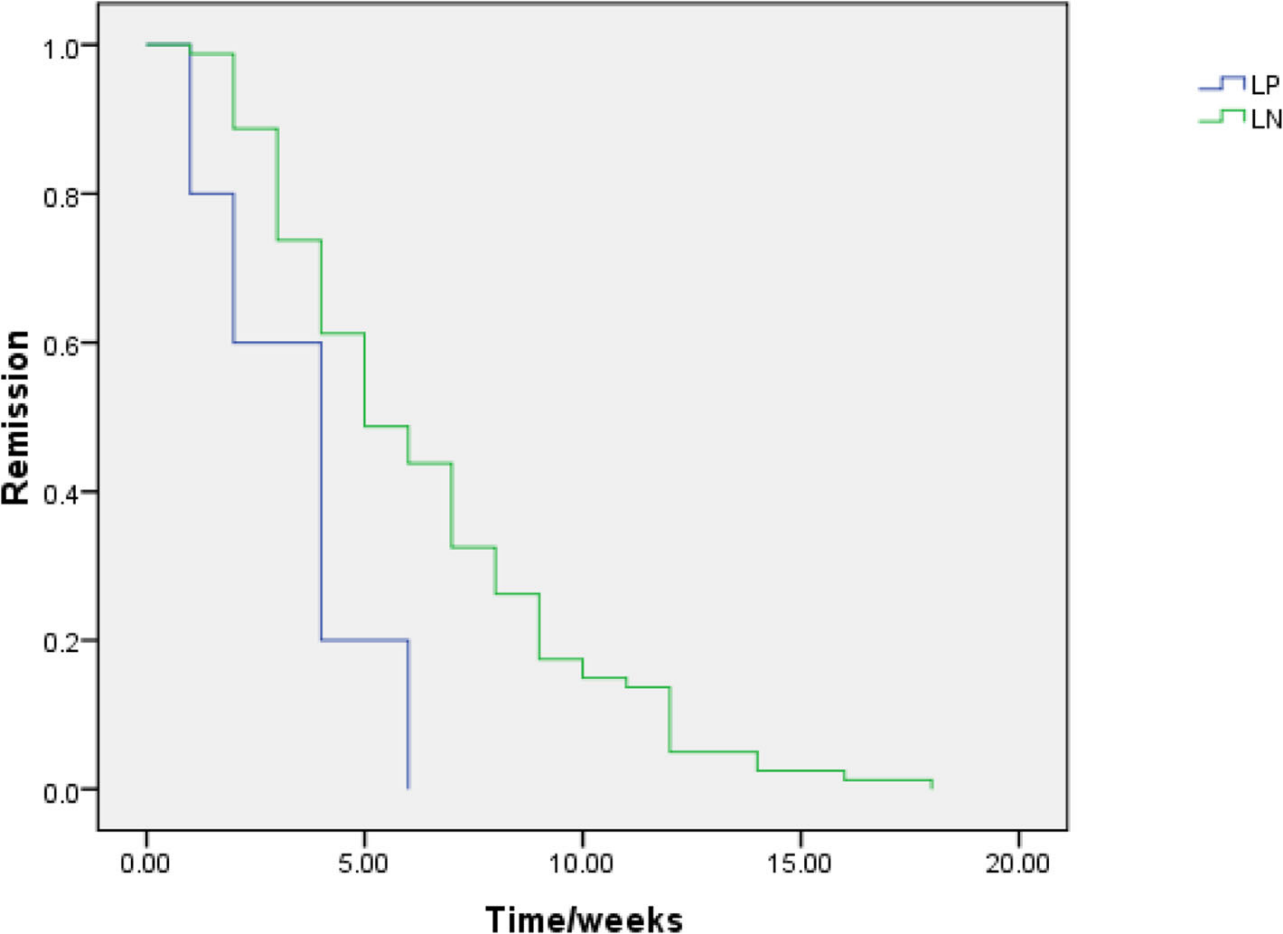
Cox-regression comparing time (weeks) for occurrence of complete remission in the two groups

*The demographic and laboratory data* (Table 1) of the 86 p-SLE studied patients with renal affection (61.43%) of all diagnosed lupus children show that: In group I (LN cases) Female to male ratio was (F/M: 7.8/1), the mean age at diagnosis was (12.24 ± 3.31y), SLEDAI was (27.13 ± 19.968). In group II (LP cases): Female to male ratio was (F/M: 7/0), the mean age at diagnosis was (13.60 ± 2.30y) with significant lower SLEDAI (17.20 ± 6.38) than the LN group. Seven cases of LP were presented with edema, four of them had hypertension. The 24 h urinary proteins (3.41 ± 0.30 g) in LP group were significantly higher than the LN group (1.10 ± 0.79 g). Significantly lower Anti-ds-DNA (230.80 ± 66.44 IU/ml) and higher C3 (75.40 ± 12.70 mg %) and C4 (13.60 ± 5.68 mg %) were noticed when compared to the LN group’s results. *Renal biopsies results* (Table 2) Group I: LN classes III, IV and III/IV were the commonest (40.70, 20.93 and 15.12%) respectively. Group II: Five cases showed MCD (5.81%) with normal LM and effaced podocytes with no immunological deposits in EM examination **(**Fig. 1**)**, two cases (2.32%) had FSGS in LM examination with > 80% effaced podocytes without immune deposits in EM examination (Fig. 2). Response to steroids was satisfactory in LP patients with short complete remission time (3.40 ± 1.95 weeks) but with more frequent relapses when compared to the LN patients as shown in Table 3 and Fig. 3. LN class II showed a shorter duration for remission on low dose steroids without relapse when compared to LP patients (Table 4).

**Table 1:** Demographic and laboratory data

	Group I Lupus Nephritis (LN) cases ***n*** = 79	Group II Lupus Podocytopathy (LP) cases ***n*** = 7	Test	***P***-value
**Age (years)** (Mean ± SD)	12.24 ± 3.31	13.60 ± 2.30	t: 1.069	0.24
**Female %** (n%) **F/M**	88.6% 7.8/1	100% 7/0	X^2^: 0.608	0.71
**SLEDAI** (Median, IQR)	22 (15–27)	16 (12–22)	U: 48.182	0.001*
**Serum creatinine (mg %)** (Mean ± SD) (Normal: 0.3–0.7 mg %)	1.05 ± 0.53	1.66 ± 0.67	t: 5.488	0.001*
**24 h urinary proteins (g)** (Mean ± SD) (Normal: < 0.15 g)	1.10 ± 0.79	3.41 ± 0.30	t: 6.324	0.001*
**C3 (mg %)**(Mean ± SD) (Normal: 90–160 mg %)	47.0 ± 10.13	75.40 ± 12.70	t: 24.611	0.001*
**C4 (mg %)**(Mean ± SD) (Normal: 10–40 mg %)	4.25 ± 2.90	13.60 ± 5.68	t: 70.240	0.001*
**Anti-ds DNA (IU/ml)** (Mean ± SD) (Normal: 30–70 IU/ml)	423.75 ± 43.20	230.80 ± 66.44	t: 3.672	0.002*

*t* t-test, *X*^*2*^ Chi-square test, *IQR* Interquartile range, *U* Mann Whitney test,

*P*-value < 0.05 is significant*

**Table 2:** Histopathological results of the taken renal biopsies

Renal biopsy histopathology		N %	
**Group I (LN)** **ISN-RPS classes**	**LN II**	6 (6.98%)	**79 (91.86%)**
	**LN III**	35 (40.70%)	
	**LN III/IV**	13 (15.12%)	
	**LN IV**	18 (20.93%)	
	**LN IV/V**	4 (4.65%)	
	**LN V**	3 (3.49%)	
**Group II (LP)** **Podocytopathy**	**MCD**	5 (5.81%)	**7 (8.14%)**
	**FSGS**	2 (2.32%)	
**Total**		**86 (100%)**

**Table 3:** Treatment outcome

Treatment outcome	Group I (LN) cases n% = 79 (100%)	Group II (LP) cases n% = 7 (100%)	Test	***P***-value
**Treatment**				
Current steroid dose (mg/day)				
(Mean ± SD)	20.12 ± 16.70	15.00 ± 7.40	t: 0.340	0.02*
Hydroxychloroquine (n%)	75 (94.9%)	7 (100%)		
MMF (n%)	46 (58.2%)	3 (42.9%)	X^2^: 3.124	0.014*
Cyclophosphamide (n%)	12 (15.2%)	0		
Azathioprine (n%)	10 (12.7%)	2 (28.6%)		
Cyclosporine A (n%)	4 (5.1%)	2 (28.6%)		
**Time of complete remission (weeks)** (Mean ± SD)	9.50 ± 6.56	3.40 ± 1.95	t: 9.327	0.001*
**Number of relapses** (Median, IQR)	0 (0–1)	1 (0–3)	U: 0.564	0.001*

*t* t-test, *IQR* Interquartile range, *U* Mann Whitney test

*P*-value < 0.05 is significant*

**Table 4:** Comparison between LP and LN class II

Treatment outcome	LN class II n% = 6 (100%)	LP n% = 7 (100%)	Test	***P***-value
**Treatment**				
Steroid dose (mg/day) (Mean ± SD)	10.35 ± 4.2	15.00 ± 7.40	t: 1.89	
MMF (n%)	0	3 (42.9%)		0.001*
Cyclophosphamide (n%)	0	0	X^2^: 0.687	
Azathioprine (n%)	3 (50%)	2 (28.6%)		
Cyclosporine A (n%)	0	2 (28.6%)		
**Time of complete remission (weeks)**				
(Mean ± SD)	2.90 ± 1.34	3.40 ± 1.95	t: 23.50	0.08
**Number of relapses** (Median, IQR)	0	1 (0–3)	U: 0.012	0.001*

*t* t-test, *IQR* Interquartile range, *U* Mann Whitney test

*P*-value < 0.05 is significant*

## Discussion

This study presented the histopathological classes of 86 p-SLE patients with renal affection. Female to male ratio were 7.8:1 in the LN group and 7:0 in LP group that is consistent with the ratio provided by many studies on children, which found a ratio of 5:1 to 8:1 [11–16]. Seventy-nine cases (91.86%) showed different classes of LN according to (ISN/RPS) grading system [10] “LN II (6 cases 6.98%), LN III (35 cases 40.70%), LN III/IV (13 cases 15.12%), LN IV (18 cases 20.93%), LN IV/V (4 cases 4.65%), and LN V (3 cases 3.49%)”. Seven cases (8.14%) had a podocytopathy morphology “MCD (5 cases 5.81%) and FSGS (2 cases (2.32%)”. In 2002, Dube et al. [17] and Hertig et al. [6] were the first to described small series of adult SLE (a-SLE) with nephrotic syndrome and biopsy findings of MCD or FSGS. In 2005, Kraft et al. [18] reported eight additional a-SLE patients with nephrotic syndrome and light microscopic findings of MCD, FSGS, or mesangial proliferative GN. Hu et al. [19] presented 50 patients with lupus podocytopathy from a 14-year biopsy registry (2000–2013) representing 1.3% of all LN biopsies. The development of nephrotic-range proteinuria in SLE without peripheral immune aggregate deposition or endocapillary proliferation on renal biopsy is a manifestation of SLE than the coexistence of idiopathic minimal-change glomerulopathy and SLE [20] with concomitant apolipoprotein L1 (APOL1) nephropathy [21]. A glomerular permeability factor released during SLE flaring due to dysregulated T cells is considered an important clue to understanding the relationship of idiopathic nephrotic syndrome and SLE [20–24]. Unfortunately, the commonly used (ISN/RPS) classification of LN does not include lupus podocytopathy [20]. *Simple criteria to diagnose lupus podocytopathy are* (1) lupus patient with nephrotic syndrome, (2) diffuse and severe foot process effacement and (3) the absence of subendothelial or subepithelial immune deposits [20]. Up till now, the frequency, prognosis and treatment of LP are not well established in p-SLE except for one case report of Ito et al. [25] who reported an 11-year lupus girl who developed steroid-resistant nephrotic syndrome (SRNS) at disease onset and her histological findings were consistent with LP. LP represents 8.93% of renal pathology in our patients, which is consistent with other reports [6, 17–19]. LP patients had a high serum creatinine with heavy proteinuria and less disease activity index (SLEDAI) than LN patients, that comes in agreement with other studies [26–28]. Patients with proliferative LN should receive more aggressive therapies rather than patients with LP [29]. In this study, the seven LP cases responded rapidly to steroids therapy and achieved complete remission within 1–3 weeks with a median time of 2 weeks. However, three cases experienced one or multiple renal relapses. Similar findings were reported in multiple studies [20, 28, 29]. Those three patients when relapsed, had an extra-renal and serological activity that supports the idea that SLE is the cause rather than an association. We repeated the renal biopsy for two cases of LP during relapse and the results were the same as the previous biopsies so the recurrence was not related to pathological transformation. The rapid response to glucocorticoid, high response rate, and high relapse rate shown by LP patients are similar to children with idiopathic MCD.

## Conclusions

- Lupus podocytopathy is rarely described in p-SLE with renal affection and its pathophysiology is still unclear without immune deposits that may be related to T cell dysfunction, which is the pathogenesis of idiopathic NS in children that makes LP a clinicopathological pattern that behaves like it.
- We think LP is a spectrum of SLE, not an association as it is related to disease activity and its initial presentation but to add LP as a class in ISN/RPS, needs more studies and larger sample size for an accurate evaluation and documentation of its pathogenesis.

## Data Availability

The datasets used and analyzed during the current study are available from the corresponding author on reasonable request.
